# Systematic Methods to Resolve Lineage-Specific Stress States in Early Mammalian Embryos and That May Enable Miscarriage Prediction

**DOI:** 10.3390/cells15110996

**Published:** 2026-05-28

**Authors:** Ximena L. Ruden, Campbell Coddington, Lynessa Asplund, Anjie Dinakin, Awoniyi O. Awonuga, Douglas M. Ruden, Steven J. Korzeniewski, Lijun Zhang, Elizabeth E. Puscheck, Daniel A. Rappolee

**Affiliations:** 1 Department of Obstetrics and Gynecology, C. S. Mott Center for Human Growth and Development, Wayne State University, 275 East Hancock Street, Detroit, MI 48201, USAanjie_dinakin@wayne.edu (A.D.); aawonuga@med.wayne.edu (A.O.A.); douglasr@wayne.edu (D.M.R.); skorzeni@med.wayne.edu (S.J.K.); epuschec@med.wayne.edu (E.E.P.); 2 Reproductive Stress Inc., Grosse Pointe Farms, MI 48236, USA; 3 Department of Pharmaceutical Chemistry, University of California, San Diego, CA 92093, USA; 4 Department of Emergency Medicine, Wayne State University School of Medicine, Detroit, MI 48201, USA; 5 Department of Population & Quantitative Health Sciences, Case Western Reserve University School of Medicine, Cleveland, OH 44106, USA; lxz759@case.edu; 6 InVia Fertility, Chicago, IL 60618, USA; 7 Department of Physiology, Wayne State University, Detroit, MI 48201, USA

**Keywords:** transcriptomic analyses, stress, developmental stem cells, trophoblast stem cells (TSCs), embryonic stem cells (ESCs), miscarriage, systematic stress classification and prediction, high-throughput screening (HTS), differentially expressed gene (DEG), NOAEL, LOAEL, IC50, gene ontology

## Abstract

**Highlights (see Abbreviations section for all acronym use, at the end of the text):**

**Part 1. Methodological framework**

- High-throughput screening defines biologically equivalent stress doses, enabling stem cell transcriptomic markers at these doses to serve as training sets for human clinical test marker sets.
- NOAEL/LOAEL frameworks enable cross-stressor comparisons at equivalent biological doses, where stem cell transcriptomic programs resolve shared homeostatic stress challenges.

**Part 2. Analytical integration and biological inference**

- RNA-seq identifies functional stress response programs in stressed stem cells and embryos, analyzed using ORA–FEA for significance and GSEA for expanded, rank-based enrichment across larger gene sets.
- Integrated analysis links stress responses to developmental outcomes, enabling inference of mechanisms underlying embryo growth delay, and implantation failure miscarriage.

**Abstract:**

Early mammalian embryos are highly sensitive to environmental, metabolic, hormonal, and genomic stress, yet embryo assessment during **I**n **V**itro **F**ertilization (**IVF)** relies largely on morphology and ploidy for embryo assessment, but these tests incompletely predict miscarriage. We present a transcriptomics based framework to classify and quantify lineage-specific stress in early embryos by benchmarking human preimplantation embryos against dose-, time-, and quality-dependent stress programs defined in **E**mbryonic and placental **T**rophoblast **S**tem **C**ells (**ESCs, TSCs**) from the implanting blastocyst. Human embryos and stressed ESCs and TSCs are screened using transcriptomic markers from eleven biologically distinct stress **G**ene **O**ntology (**GO**) groups that define functional stress states and enable quantification of pathway presence and upregulation, pathway activity, and downstream outcomes. This framework determines whether the **I**ntegrated **S**tress **R**esponse (**ISR**), once initiated, resolves to enable the **D**evelopmentally **A**ssociated **S**tress **R**esponse (**DASR**). **H**igh-**t**hroughput **s**creening (**HTS**) titrates stress to define increasingly risky yet biologically equivalent doses for levels of diminished stem cell growth across mechanistically diverse stressors. Then bulk RNA seq derives lineage specific transcriptomic markers putatively respond to common levels of diminished growth and that distinguish weak vs. strong stress and resolved vs. unresolved ISR. These stem cell transcriptomic signatures are applied to bulk RNA seq data from IVF embryos graded for morphology or adhesion, enabling quantitative inference of stress burden, lineage vulnerability, and developmental trajectory.

## 1. Introduction

### 1.1. Key Concepts in a New Complementary Framework for Existing Stress Risk Assessment

1. Quantitative stress biology should be integrated with developmental logic.
2. Introducing a novel experimental–computational workflow.
3. Positioning an experimental workflow framework as *the* bridge from stress risk assessment in toxicology → embryo stress (and stem cell) risk assessment that may enable optimization predictions in reproductive and regenerative medicine.

### 1.2. Introduction: Biology

Somatic cell stress homeostatic baselines are retrospective and developmental stem cell baselines are prospective.

Stress in developmental stem cells is not the same as stress in differentiated somatic cells in adults. While both share dose-response mechanisms, these occur only at early stress onset [1]. Developmental stem cell stress homeostasis is prospective. It supports functions that must emerge within defined developmental timelines. The stress homeostatic target requires sufficient cell numbers. These arise through proliferation and differentiation of tissues not yet formed at stress onset. Both developmental and somatic stress responses preserve cellular homeostasis. They do so through dose-dependent activation of sensor–effector gene pairs. These prospective mechanisms remain speculative and are discussed here in the context of IVF stress analysis The greatest risk in IVF is miscarriage, which is also the most common outcome for fertilized embryos. Understanding miscarriage mechanisms in IVF informs broader mechanisms of pregnancy loss. This article presents a methodological framework that adapts toxicological dose–response and transcriptomic strategies to quantify stress in early embryos.

While this framework draws on concepts from toxicology, developmental biology, transcriptomics, and clinical IVF, its primary purpose is methodological: to adapt established toxicological strategies—specifically high-throughput screening (HTS) and biologically equivalent dose frameworks and their associated transcriptomic responses—to the analysis of developmental stress in early embryos. Developmental and clinical applications are presented as use cases that illustrate how this framework can be applied across systems, rather than as independent domains of equal scope.

**Core (center of the framework presented here)**

- Toxicology → HTS → biologically equivalent doses → associated transcriptomic programs;
- Transcriptomics → stress programs → ISR→DASR time-dependence, and dose- and quality-dependent programs and markers;
- Output → predictive embryo stress framework applicable across many applications benefitting from a fundamental understanding of developmental stress.

**Applications using core frameworks (not co-equal domains)**

- IVF/embryo selection: primary application infertile women, assessing stress in the conceptus in fertile women (secondary application), where the likely focus is qualitatively strong stress markers;
- Developmental biology: the three stem cell lineages of PrE, TSCs, ESCs and the mechanistic validation of stress homeostasis and adjustment of normal developmental trajectories;
- Toxicology/NAMs: origin of methods, defining stress effects persisting from pregastrulation embryogenesis into axis formation during gastrulation;
- Regenerative medicine: extension/future use, optimizing huESC, and huiPSC isolation and maintenance, where the likely focus is qualitatively weak stress markers.

Sensor–effector transgenes report proportional stress responses across seven defined stress categories. These were proposed by a committee established by the National academy of Sciences that expanded to ten with later studies (Eleven stress categories define functional axes of stress biology here (Supplementary Table S2). The categories presented in this study are derived from prior frameworks and refined for developmental systems [2,3,4] and were created by the authors as a means to understand and measure development and reproductive stress based on the literature of mechanisms and markers used during stress homeostasis in developmental stem cells [1,5,6].

### 1.3. High-Throughput Screening Establishes Biologically Equivalent Stress Doses—Responses at These Doses Are Defined by Associated Transcriptomic Programs

**Figure 1:**
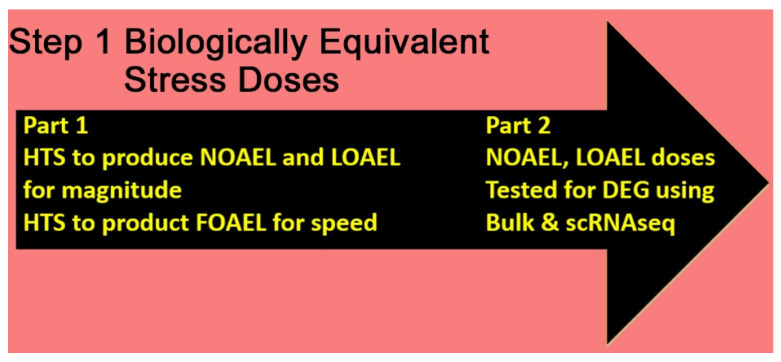
Workflow for identifying biologically equivalent stress doses and corresponding transcriptomic responses in ESCs and TSCs. High-throughput screening (HTS) defines dose- and time-dependent growth suppression, which is used to establish NOAEL, LOAEL, and IC50 doses. Cells are then re-cultured at these defined doses for transcriptomic analysis to identify stress-response programs at these biologically equivalent stress doses.

Reporter transgenes enable dose- and time-dependent stress testing in **T**rophoblast **S**tem **C**ells (TSCs) and **E**mbryonic **S**tem **C**ells (ESCs) high-throughput screens (HTSs) (Supplemental Table S1) [1,4,7,8,9,10,11,12,13]. Here we redo the toxicology risk categories proposed previously [4] (Supplemental Table S1) to address concerns of stress from IVF and regenerative medicine (Supplemental Table S2). Re-testing TSCs and ESCs defines transcriptomic programs and identifies shared and unique markers across stressors (Figure 1) to enable an understanding of the functions of transcriptomic programs used. In addition, their shared and unique marker genes across diverse stressors can be binned into distinct dose-, time- and quality-specific marker groups [5,13,14,15], unpublished data]. These groups may enable prediction of miscarriage or sublethal stress effects in human embryos and stem cells.

### 1.4. The Period of Maximal Miscarriage Reflects the Difficulty of Meeting Developmental Demands Under Stress

Proliferation, necessary for early embryo survival, is hard to maintain and becomes harder with stress. During early, post-fertilization and embryo development, there is the most rapid proliferation of TSCs and ESCs compared with any stem cell in the life history of the organism [1,6]. Mouse and human TSC and ESC growth acceleration occurs at implantation and TSC population size and acceleration precede that of ESCs. Sufficient proliferation enables downstream parenchymal function. We use stress-diminished proliferation as a baseline. This defines biologically equivalent stress doses for downstream analysis (Figure 1).

Sufficient differentiation to certain lineages is also required for early embryo survival. Early embryos must produce sufficient endocrine signals to communicate with maternal tissue. This occurs despite extreme size differences between the conceptus and maternal blood volumes. **H**uman **C**horionic **G**onadotropin (**hCG)** must be secreted at sufficient levels to support endometrial transition [1]. hCG levels must increase exponentially until placental support is established.

Proliferation and differentiation together define stress outcomes. These processes govern developmental responses during stress homeostasis. They occur between mutually exclusive programs of, specifically, aerobic glycolysis supporting proliferation vs. oxidative phosphorylation and mitochondrial metabolism supporting differentiated parenchymal function. Additional competing demands arise during stress adaptation. These include shifts from anabolic to catabolic programs.

### 1.5. Stress Diminished Growth and Homeostatic Compensatory and Prioritized Differentiation Requires an ISR–DASR Shift Soon After Stress Initiates

Reduced proliferation increases compensatory differentiation responses. This can override stemness-maintaining programs. But dose- and quality-dependence are key aspects of how the increased differentiation plays out. There are two broad categories of stressors, weak and strong, that affect the early embryo in very different manners for all sublethal doses [5]. For weak stressors, lineage balance is preserved at all doses, with stemness overridden to emulate and sustain normal differentiation (ND). Strong stress imposes a lineage bias toward the first lineage. This bias increases as stress intensifies. Thus, immediately after stress onset, the primary goal is to ensure cellular survival, followed by divergence into distinct developmental trajectories determined by stress strength and stress quality (i.e., weak vs. strong categories of stress).

**Figure 2:**
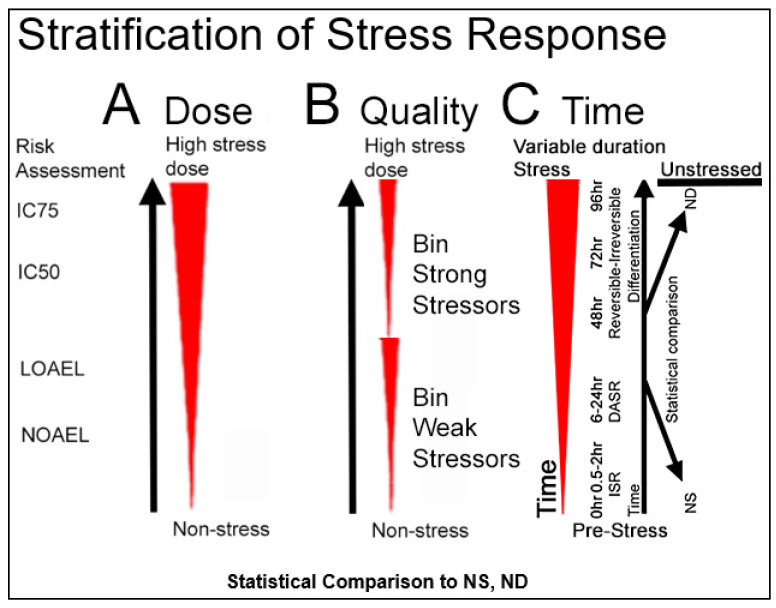
Transcriptomic analyses of ESCs and TSCs reveal dose- (**A**), quality- (**B**) and (**C**) time-dependent stress markers that enable analyses of other reports of incompletely understood transcriptomic markers for medically important stem cells, embryoids, and embryos where an understanding of optimization is needed. The irreversible ND of TSCs requires 48 h whereas the override of TSC stemness and then differentiation by a strong stress requires 96 h to become irreversible (**C**). The hypothetical ISR to DASR shift was predicted in ESCs based on sequential unique shared pan-stress marker sets immediately after the initial stress response (ISR) and then a following developmental planned parenchymal milestones [5] and is shown in TSC timing to be between 0.5 and 24 h [unpublished data]. The X in the red isosceles triangles and shown in TSC timing between 0.5 and 24 [unpublished data]. In (**B**), weak and strong stressors are binned in distinct groups, as there is a qualitatively distinct discontinuity between them [5]. **NS** = normal stemness and **ND =** normal differentiation (by removing growth factor in culture media that maintains NS).

The initial post stress period is the **I**ntegrated **S**tress **R**esponse (**ISR**, Figure 2). ISR must resolve rapidly to enable DASR. This transition restores energy balance and supports the **D**evelopmentally Associated **S**tress **R**esponse (**DASR**) unpublished data]. The DASR supports a stress-induced emulation of stressless ND. Embryo survival is best when DASR starts as quickly as possible so to emulate ND as closely as possible.

Normal development, proliferation, and differentiation are intrinsically challenging, especially during early embryogenesis when developmental demands are difficult to meet. The high rate of human embryonic loss—estimated at ~70% miscarriage [1,6]—reflects this fundamental constraint. Stress both intensifies these challenges and activates adaptive, lineage-specific responses in TSCs and ESCs. These mechanisms reflect evolutionary selection for reproductive success under stress. They date back to early eutherians such as Juramaia sinensis, the “Jurassic mother from China” [14]. Studies of stem cell type-specific stress adaptations to dose-, time-, and quality-dependent stress identify evolutionarily selected hemostatic transcriptomic programs that enable a modicum of success during this early difficult developmental period prone to miscarriage.

In summary, early embryo stress biology must be interpreted in the context of its growth objectives. Early embryos are most sensitive to stress during their periods of mandatory exponential growth. In addition, during those key periods, essential differentiated-stem-cell-specific lineages must be allocated to meet distinct quantitative milestone deadlines. Therefore, during stress homeostasis, proliferative expansion followed by emulation of the normal sequence of differentiation is required for survival. A key concept is that early human embryos miscarry, or suffer sublethal adverse effects, not because they encounter stress, but because stress is unresolved during their most sensitive periods of mandatory exponential growth and precluding essential differentiated function. Therefore, stress biology must be interpreted in the context of lineage-specific proliferation demands and quantitative functional milestones requiring proliferative expansion followed by emulation of normal sequence of differentiation during stress homeostasis.

### 1.6. Introduction: Methodologies

Current embryo assessment in IVF is based primarily on morphology, morphokinetics/time-lapse, and selected genetic testing, whereas transcriptomic and stress-informed methods remain emerging research approaches. Developmental toxicology increasingly uses integrated HTS approaches rather than relying on a single model. Regenerative medicine lacks optimal conditions for maintaining pluripotent stem cells. These limitations introduce epigenetic errors [15,16]. Here we propose complementary new emerging, methodologies intended to provide hypothesis generating stress discovery and additional orthogonal dimensions to existing methods in each of these three areas of medicines addressing or using reproductive and developmental models.

This report provides an overview of methodologies for generating transcriptomic markers specific to developmental stem cell types. It also details the use of these markers, in conjunction with Gene Ontology (GO) stress function marker groups and ad hoc TSC and ESC progeny lineage markers, to assess time-dependent, dose-dependent, and qualitative stress responses. These may be used to predict levels of stress, assess likelihood of outcomes for IVF embryos and embryoids, and how to optimize pluripotent stem cells in regenerative medicine. Analysis of markers sets should suggest mechanisms and thus methods to mitigate or obviate stress.

### 1.7. Introduction: Novel Contributions of This Work

This framework integrates toxicology, developmental biology, transcriptomics, and IVF research as a unified methodological approach. To clarify the distinction between prior knowledge and new contributions we summarize below the specific advances introduced in this work:

1. **Structured stress taxonomy:**

While individual stress pathways (e.g., ISR, ROS, mitochondrial stress) are well established, we organize these into eleven functional categories that capture progression from stress sensing to downstream outcomes. This structured taxonomy provides a practical framework for systematic analysis across datasets.

2. **Integration of toxicological dose frameworks with developmental biology:**

We adapt high-throughput screening (HTS)–derived biologically equivalent dose concepts (NOAEL, LOAEL, IC50) to developmental stem cell systems, enabling comparison of diverse stressors at equivalent functional impact. This enables definition of “pan-stress” markers common to dose- or quality-dependent stress and defined more broadly as markers that mark qualitative differences in weak vs. strong stress.

3. **Lineage-resolved stress interpretation:**

We introduce a two-stage analytical approach in which Gene Ontology (GO)–based stress categories are filtered through experimentally defined embryonic and trophoblast stem cell transcriptomes to achieve lineage-specific resolution.

4. **Quantitative stress indices (SDRI, SQRI, STRI):**

We propose three indices that summarize stress magnitude, qualitative response type, and temporal resolution dynamics, enabling quantitative comparison across samples. These indices may work well during analysis of single clinical samples.

5. **Application to IVF embryo assessment:**

We extend this framework to human embryo transcriptomic datasets, proposing that stress-resolved molecular signatures may complement morphology-based embryo grading and improve risk stratification.

6. **Hypothesis-generating integration across domains:**

Rather than introducing new biological pathways, this work provides a conceptual and computational framework that integrates existing knowledge into a testable system for studying developmental stress and its clinical implications.

## 2. Stepwise Identification of Biologically Equivalent Responses to Stress-Diminished Stem Cell Growth Defining Transcriptomic Responses of Diverse Stressors, Enabling Cellular and Developmental Homeostasis

### 2.1. Step 1 in Creating Systematic Stress Markers Starts with Part 1—An HTS Is Used to Produce Biological Equivalent Stress Risk Doses, and Part 2—To Produce Transcriptomic Programs for These Doses the to Assess Stress Risk

Step 1 in systematic analysis of stress in stem cells and embryos is to examine diverse stressors (Figure 1). In **Part 1,** HTS is used to determine biologically equivalent stress risks for dose- and dependent adverse effects. Those need to be determined in order to separate outcomes due to stress strength vs. quality. Systematic stress analysis begins with diverse stressors. These are used to define biologically equivalent response. In **Part 2**, gene expression for those doses can be examined with RNAseq. This enables an understanding of transcriptomic programs used, and their shared and unique marker genes across diverse stressors that can be binned into distinct dose-, time- and quality-specific marker groups [5,13,17], unpublished data]. These groups should enable prediction of miscarriage or sublethal stress effects in human embryos and stem cells.

*Time is as important as dose in understanding the stress responses of developmental stem cells.* While examining biologically equivalent stress doses can help to separate effects due to stress strength vs. quality, there is an additional third axis by which stress responses can be stratified: time (Figure 2). It should be clear that dose-, stress- and quality-dependent outcomes are orthogonal dimensions. These three dimensions define not stress magnitude alone, but the probability of developmental recovery for the outcomes of proliferation, stem cell depletion, and lineage-insufficiency.

### 2.2. Step 2 the Systematic Analysis of Stress in Stem Cells and Embryos Is Enabled by Comparing Gene Expression (DEG) Data of Stressed Stem Cells, GO Groups and Relevant Clinical Data and Models of Clinical Data (Figure 3)

**Figure 3:**
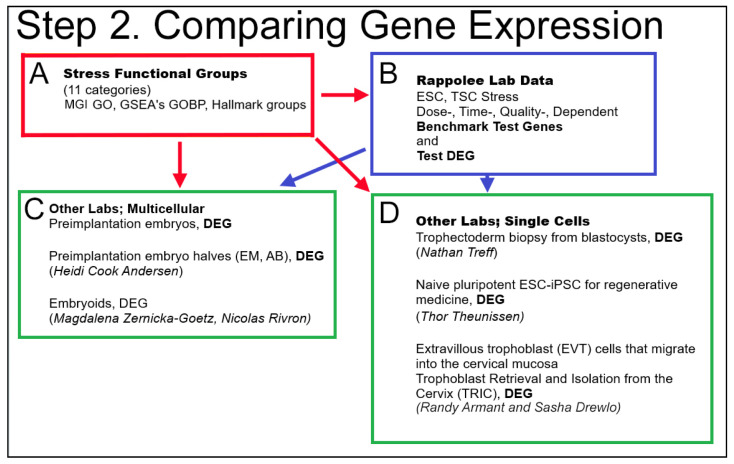
Eleven stress transcriptomic benchmark marker gene categories (**A**) are a framework to be used to filter isolated, stressed stem cell time-, dose-, and quality-dependent DEG markers (**B**), directly for embryos or embryoids (**C**), or it could benchmark any of the potentially stressed stem cells from stem cells isolated from embryos, embryoids, or cultured stem cells (**D**)**.** (**A**,**B**) are test marker sets from many GO groups for different categories of stress (see supplemental Table S2), and stressed stem cell for dose-, time-, and quality-dependent stress. (**C**,**D**) are test sample transcriptomic markers of embryos and embryoids (**C**) and stem cells (**D**). DEGs are marker sets screened by test gene sets, DEGs are experimental transcriptomes to be screened, and genes are the established marker sets used to screen. The best method to use as screening benchmarks from (**B**) are direct stem cells stress marker genes or stress marker sets pre-screened and filtered with the benchmark genes from the 11 stress categories (Supplemental Table S2, bottom of (**B**)). Citations for (**B**) from the Rappolee lab [5,17], unpublished data], for (**C**) from the Heidi Cook-Andersen lab [20], Zernicka-Goetz and Rivron labs [21,22], and for (**D**) from the Treff lab [23,24], Theunissen lab [25,26,27], Armant and Drewlo labs [28,29,30,31].

Stress in embryos, embryoids, and isolated ESCs and TSCs (Figure 3C,D) can be queried directly using GO stress gene sets, or indirectly by first resolving stress programs in lineage-defined stem cells (ESCs and TSCs) across dose, time, and qualitative adverse effects severity. Pre-filtering GO stress genes through stem-cell-specific stress transcriptomes yields lineage-restricted marker sets that increase biological specificity when applied to bulk embryo RNA-seq as a screen or used to inform the outcomes of GO group marker screens of embryo markers. This two-stage filtering enables discrimination between generic stress activation and lineage-specific developmental stress responses that underlie implantation failure. However, smaller prefiltered marker sets can be less useful. Direct testing of human embryos and other samples can be done with both stress GO groups, and lineage-specific stress markers. In addition, GO group testing of stressed TSC and ESC lineages can be used to inform stem-cell-type-specific stress program usage when separately analyzing GO group marker analysis of embryos.

Before looking at individual genes, it is best to examine stress markers en masse, as a means to define the stress level and rate of stress resolution from the changing shape of the gene expression databases. Significant DEGs can be used to determine the proportion of DEGs vs. all expressed genes, and fraction of up- vs. down-regulated DEGs. These criteria make up the first stage of identifying the proposed, sequential ISR vs. DASR stress periods [5,12], unpublished data].

This review is in four parts, followed by Supplemental Table S2, which includes eleven types of GO stress marker sets. There are an additional two Tables (Supplemental Tables S3 and S4) of ad hoc developmental lineage markers for: extraembryonic ectoderm (i.e., TSC) lineages, e**X**tra-**E**mbryonic e**N**doderm (aka **XEN**) lineages, and the three stages of ESC lineage pluripotency (naïve, formative, and primed).

## 3. Before Going into the Four Parts of Stem Cell Stress Analysis, Ad Hoc Stressed Stem Cell Markers, as Well as Defined Stress GO Groups, Will Be Defined

We organize embryo-relevant stress into *eleven functional categories* (Supplemental Table S2, Figure 3). The selection of eleven stress categories reflects a pragmatic and structured representation of major functional axes of cellular stress, spanning stress sensing, signal integration, and downstream outcomes. These categories were derived from prior toxicological and stress-response frameworks and refined to capture distinct but interacting biological processes relevant to early development. While the number eleven is not uniquely determined, it represents a balance between biological resolution and analytical tractability, enabling systematic comparison across datasets without excessive fragmentation or redundancy.

The eleven stress categories are not redundant; they are ordered to reflect escalation from adaptive sensing to irreversible damage in each category. These categories are not a list but a taxonomic relationship; it is a structured, hierarchical classification system that organizes functions into logical categories and subcategories based on shared properties, such as purpose or behavior. In addition, it is likely that different stressors will induce unique, as well as shared, outcomes reported by the 11 categories (Supplemental Table S2) in stressed TSC and ESC transcriptomic markers (Figure 1 and Figure 2), commensurate with dose-, time- and quality-dependent stress. In addition, the 11 categories can better define TSC and ESC stress responses. Stress category markers can pre-tune lineage-specific stress markers prior to testing human embryos, embryoids, or isolated stem cells or either can be used directly to test quantitative and qualitative stress outcomes in test samples. The relationship of the 11 categories is not simply as a list but as a taxonomic integration of ISR stress response that when resolved, hypothetically enables blossoming of DASR related transcriptomic markers sets and functions in ad hoc developmental lineages, and their marker defined outcomes are shown in Supplemental Tables S3 and S4.

(1) Pan-stress protein kinase signaling; **AMP**-activated **P**rotein **K**inase (**AMPK**) vs. **S**tress-**A**ctivated **P**rotein **K**inase (**SAPK**);
(2) Stress granule dynamics;
(3) **R**eactive **O**xygen **S**pecies (**ROS**), with mitochondrial emphasis oxidative stress;
(4) Hormonal/**G**lucocorticoid **R**esponse (**GR**) stress;
(5) Aneuploidy-associated stress;
(6) Mutagenic/genotoxic stress;
(7) ISR (aka **U**nfolded **P**rotein **R**esponse or **UPR**);
(8) ER stress, protein overload;
(9) Mitochondrial/oxidative stress;
(10) ROS escalation;
(11) Aerobic glycolysis–oxidative phosphorylation switching.

For each category, we define GO–based marker sets that separate **stress-sensing-pathway presence and upregulation**, **pathway activation**, and **developmental outcomes**, allowing discrimination between reversible adaptive responses and progression to a maladaptive **DASR**.

Gene Ontology (GO) categories are inherently overlapping and context-dependent, reflecting the interconnected nature of biological processes rather than discrete, independent pathways. For example, Reactive Oxygen Species (ROS) signaling and mitochondrial stress share substantial functional overlap, and downstream outcomes such as mitophagy may arise from multiple upstream stressors. In this framework, such redundancy is not treated as a limitation but as a biologically meaningful convergence of stress-response pathways.

To mitigate false positives and overinterpretation, GO categories are not analyzed in isolation. Instead, we require concordance across related functional groups and interpret enrichment patterns in the context of pathway hierarchy (stress sensing → integration → outcome) and lineage-specific expression data derived from stem cell models. This layered approach reduces reliance on any single GO term and improves biological specificity.

## 4. Why GO Group Functional Analysis Alone Is Insufficient for Stress Adaptation by Developmental Lineages

Importantly, the initial characterization of stress responses is not derived from GO-based functional grouping, or other analyses requiring names of single genes or groups of genes, but from the global structure of the differentially expressed gene (DEG) dataset itself. Features such as the total number of DEGs, fold-change distributions, and the balance of up- vs. down-regulated genes over time provide a first order “shape” of the stress response. These properties capture orthogonal axes of stress—time, dose, and response quality—that are subsequently refined through functional annotation. Thus, GO-based analyses represent a second layer of interpretation built upon this primary transcriptomic structure. The first layer of total significant DEGs, FC range of significant DEGs of stressed vs. unstressed differentiating cells, and total number of GO groups is the first analysis as previously published [5,17] and serves as a framework for the analyses analyzed and discussed here.

Overlap between GO categories is handled computationally at multiple levels. First, enrichment analyses are performed using a defined background gene universe restricted to expressed genes, reducing bias. Second, related GO terms within each stress category are grouped and interpreted collectively rather than individually. Third, enrichment results are filtered through lineage-specific transcriptomic datasets (ESCs and TSCs), allowing assignment of stress responses to specific developmental contexts. Finally, convergence of multiple categories on shared downstream outcomes (e.g., mitophagy) is interpreted as evidence of integrated stress responses rather than category-specific effects.

A problem with studies of stress in early embryos and their stem cell lineage is multi-fold. (1) Many of the stem cell lineages (e.g., formative pluripotency or primitive, parietal and visceral endoderm) have no GO groups to provide lists of functions and markers. (2) Not all human TSC lineages have GO groups, but mouse has a good range of lineage-specific GO groups. (3) A systematic software to apply stress transcriptomic analysis has not been developed, although a method to systematically categorize stress into eleven categories with sensor-effector governing mechanisms has been proposed [2,18].

Here we provide ad hoc tables for the three sub-lineages of primitive endoderm (XEN, Supplemental Table S3) and for mouse TSC lineages (Supplemental Table S4). For naïve (0th lineage) and formative ESC pluripotency (2nd lineage where primitive endoderm is 1st lineage), “core” markers were described previously [5,17]. In addition, primed pluripotency was discussed previously [19]. Supplemental Tables S3 and S4 are necessary complementary means to understand stress types and their markers in relation to Supplemental Table S2.

Therefore, an intentional assignment of types of stress to types of stem cells in the early embryo is needed and Figure 3 is a systematic workflow to use GO functional group genes or stressed stem cell genes (Figure 3A,B) to identify and quantitate stressed lineage and stress type of experimental stressed embryos, embryoids, and stem cells Figure 3C,D or GO groups from the 11 stress categories (Supplemental Table S2). (Figure 3A) define types of stress in specific time- and dose-dependent TSCs or ESCs and then these stem cell specific genes are used to define these stem cell specific stress function in embryos and embryoids (Figure 3C,D), where bulk RNAseq may obfuscate this in studies such as Chousal et al. [20]. In summary, Figure 3 summarizes how a workflow summarizes how we operationalize use of the three tables integrated with the HTS results for biologically equivalent adverse effects doses and the transcriptomic programs used to adapt to stress that diminishes proliferation.

The advantage of GO groups is they can define whether a pathway is (1) upregulated and present, (2) whether it is active and (3) if it has downstream sequelae (see Supplemental Table S2). Fine-tuned GO groups for regulatory transcription factors (**TFs**) for stress or lineage are those that assay genes with known sequence specific response elements in their promoter and are thus directly regulated by the TF.

This report will go through four parts, the sequential steps in creating and applying stem cell-specific stress response markers, generic categories of stress markers, and functional GO group markers. These four parts describe a long-range workflow, coupling HTS, transcriptomics supporting biological effects from the HTS, and the analysis of these stress response programs at key risk doses and exposures.

Transcriptomic preprocessing and statistical analysis followed standard RNA-seq workflows. Raw count data were normalized using library-size-adjusted methods (e.g., DESeq2 or equivalent) and differentially expressed genes (DEGs) were identified using statistical models with multiple testing correction (false discovery rate, FDR < 0.05). ORA–FEA was performed on DEG sets defined by adjusted *p*-value thresholds, while GSEA used ranked gene lists (e.g., log_2_ fold change) without hard thresholds.

To reduce false positives, enrichment analyses were performed using appropriate background gene universes, and results were interpreted with attention to GO term redundancy and biological plausibility. Where possible, findings were cross-validated across related GO terms and integrated with lineage-specific expression data.

## 5. Part 1: Sequential Steps to Identify and Query Developmental Stem Cells Exposed to Multiple Stressors for Their Biological Equivalent Levels of Stress Risk and Produce Transcriptomic Databases, Marker Sets, Programs, and Single Gene Functions

**Figure 4:**
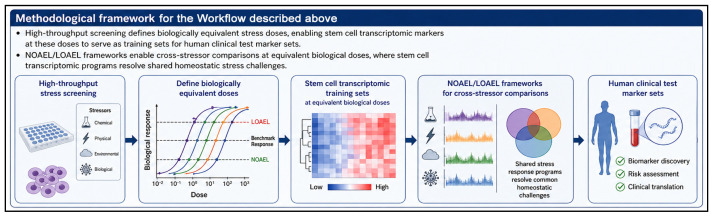
Workflow for the four sequential steps defined in Section 5, Section 6, Section 7 and Section 8 above.

*Workflow 1: High throughput screens (HTSs) establish time and dose dependence, and qualitatively different (*e.g.*, weak, and strong) and biologically equivalent stress doses for stem cells (Note that we discuss four workflow steps, and they are summarized graphically in* Figure 4. Specifically, mechanistically diverse stressors at different concentrations can cause similar levels of adverse developmental effects. Terms for biologically equivalent levels of insufficient growth caused by increasing stress, borrowed from toxicology, are NOAEL, LOAEL and IC50. NOAEL is the highest level of stress which does not significantly decrease growth (no observable adverse effect level), LOAEL is the lowest stress diminishing growth (lowest adverse effect level), and IC50 is half maximal growth suppression dose (inhibitory concentration yielding 50% growth suppression). At each of these doses the developmental stem cell must solve a biologically equivalent problem. HTSs have been used in ESCs to create qualitative and quantitative biological equivalence between doses [8,9] and in dose dependent effects in TSCs [13], unpublished data]; all of these are on adverse growth effects.

*Workflow 2: Once these are determined in the HTS, the stem cells are re-cultured at these doses and transcriptomic programs analyzed*.

## 6. Part 2: Sequential Steps to Analyze the Stress-Induced Transcriptomic Program Responses to Biological Equivalent Stress Levels Shared by Developmental Stem Cells

### 6.1. Workflow 1: Stress Transcriptomics Are Informative Without Prior Knowledge of Individual Gene Identities, Instead Leveraging Global Changes in DEG Quantity, Directionality, and Kinetics

The following five transcriptomic database metrics pinpoint stress kinetics:

***(1)** The total number of DEGs*: *p* value significant, adjusted *p* value significant, or **F**alse **D**iscover **R**ate (**FDR**).

***(2)** The level of up- and down-regulated DEG*s and the change of these ratios with resolution of the initial stress.

***(3)** The quality of stress—weak or strong*—to better understand the chance of success of embryo implantation into the uterus and later likelihood of birth.

***(4)** The kinetics of the stress response, examining genes identified as ISR and DASR* via *GO groups*.

***(5)** The total number of GO groups*, *p* value significant, adjusted *p* value significant, or FDR.

For example, these metrics can be used to determine what levels of strong stress measured by stem cell markers best correlate with clinical samples, such as poor morphology embryos from IVF. Thus, levels of strong or weak stress may be associated with implantation failure and miscarriage.

### 6.2. Workflow 2A: Filter GO Group Marker Genes Against the Time- and Dose-Dependent and Qualitatively—Different Weak vs. Strong Transcriptomic Stem Cell DEGs. This Produces Marker Sets for Each Stem Cell Type to Probe Stressed Human Embryos and Does Not Require Gene Identification

*p* value and FDR significance are used to establish stem cell stress specific marker sets to query embryos for their TSC and ESC lineage cells’ stress levels. *Another slightly more focused and detailed means to use stressed stem cell transcriptomics is to use them to query which stress GO groups arise in response to specific types of stress into eleven key stress categories*.

***(1)** Pan-stress protein kinases that include AMPK and SAPK* integrate dose-dependent proportional response to many types of stress in the moment, from minutes to hours into long-term (hours to days) developmental outcomes like proliferation and differentiation [6]. What is unique to AMPK and SAPK and a few other pan-stress PKs, compared to the entire kinome of 500 PKs shared by mouse and human, is the universal regulation of the length of the cell cycle in all mammalian cells—AMPK regulating G1 phase length [32,33,34] and SAPK S phase length [35,36]. Since cell cycle completion requires hours to days, the short term proportional stress response in the moment is projected into a long term outcome by coupling in the same stress enzyme, decreasing growth, and increasing differentiation. SAPK is important in reporting preimplantation embryo stress and its inhibitors may improve embryo development [37,38,39].

**(2)** *Stress granule* formation. This category captures types of functional stress for reversible disassembly of mRNA loaded ribosomes, examining assembly of stress granules during peak early stress and disassembly of stress granules and reassembly of mRNA loaded ribosomes as stress resolves. Stress granules tend to be an early part of the stress responses as mRNA loaded ribosomes disassemble and are stored in stress granules from 0.5 to 2 h and then disassemble and reform mRNA loaded ribosomes after stress adaptation in the ISR [40,41,42,43].

**(3)** *ROS with mitochondrial emphasis or oxidative stress response* with emphasis on mitochondrially-mediated adverse functions. This category captures mitochondria as the origin and early integrators of stress, rather than downstream oxidative damage. In early embryos and stem cells, mitochondrial membrane potential, electron transport chain (ETC) integrity, and redox buffering determine whether stress remains adaptive or escalates into failure. Mild mitochondrial ROS act as signaling intermediates that activate AMPK, mitophagy, and metabolic rewiring without global damage, whereas early collapse of mitochondrial function precedes SAPK activation, DNA damage, and apoptosis. Thus, this category assays mitochondrial competence under stress, not generalized oxidative injury, and is most informative early (minutes–hours) in ISR resolution trajectories [44,45,46,47].

**(4)** *Hormonal stress *(like cortisol). This is important to assay because in animal models hormonal stressors like cortisol and external stressors like bisphenol A (BPA) can synergize in reducing litter size [1,6]. These can also cause adverse stem cell growth effects, as are reported in the preimplantation blastocysts isolated from stressed mothers.

**(5)** *Aneuploidy *(caused by reversine experimentally [48,49]). This is important because in humans, aneuploidy is associated with a high fraction of very early miscarriage by implantation failure (no hCG detected) or chemical pregnancy loss (hCG detected but embryo lost). In humans, 70% of fertilized embryos are lost to miscarriage, 30% by implantation failure with hCG never detected, 30% miscarry during chemical pregnancy when hCG is detected begins exponential increase and then plateaus before miscarriage, and 10% clinical miscarriage after ultrasound detection of the embryo or fetus. Markers are available, and HTS covers the required time periods of TSC and ESC development, to model implantation failure and chemical pregnancy miscarriage.

***(6)**** Mutagenic stress (caused by benzo(a)pyrene*). This category captures *direct chemical damage to DNA,* distinct from oxidative, metabolic, or translational stress. Benzo(a)pyrene (BaP) is metabolized to diol-epoxides that form *covalent DNA adducts*, which have been directly detected in *human and mouse blastocysts from smoke-exposed parents* [50,51], demonstrating pre-implantation genotoxic injury [52,53,54]. Industrial pollutants such as PFAS and phthalates further induce replication stress and impair DNA repair while acting as endocrine disruptors [55,56]. In early embryos, limited DNA repair capacity and lack of lineage redundancy make even low-level mutagenic stress disproportionately likely to trigger *ATR/ATM–p53 checkpoints*, lineage depletion, implantation failure, and early miscarriage, justifying this category as a distinct stress axis [56]. This is important because DNA adducts for di-esterified BaP are detected in blastocysts from smoking parents.

**(7)** The integrated stress response (ISR)—which includes pathways such as PERK and the unfolded protein response (UPR)—is a central regulator of translational control, not merely a component of endoplasmic reticulum stress [57,58,59]. Activation of PERK leads to phosphorylation of eIF2α, rapidly reducing global protein synthesis while selectively promoting translation of stress-adaptive genes, including those regulated by ATF4. In embryos, ISR activation is typically transient and protective, supporting survival during acute stress and coordinating with stress granule dynamics. However, when ISR activation is prolonged, it induces CHOP and contributes to developmental failure, likely preventing the DASR that hypothetically follows a resolved ISR. Thus, the ISR functions as a translational decision point, determining whether cells pause and adapt or progress toward failure. This helps explain why ISR inhibition can improve embryoid development even without correcting upstream stressors. Mechanistically, the ISR is closely tied to cellular metabolism, protein synthesis, and ribosome function. This is particularly relevant to the CEPT small-molecule cocktail—which includes Chroman 1, Emricasan, polyamines, and trans-ISRIB—where the “T” component (trans-ISRIB) acts as an ISR inhibitor [57,59,60,61] and enhances embryoid development during peri-implantation-like culture conditions. The ISR is a *central translational control system*, not simply “ER stress.” PERK-mediated eIF2α phosphorylation rapidly suppresses global translation initiation while selectively inducing ATF4-regulated stress adaptation genes. In embryos, ISR activation is often *protective and transient,* enabling survival during acute stress and coupling to stress granule dynamics. However, prolonged ISR drives CHOP induction and developmental failure (i.e., hypothetical blocked DASR). This category therefore reports *decision-making at the translation level*—whether cells pause and adapt or commit to failure—and explains why ISR inhibitors (e.g., the “T” in CEPT) can improve embryoid development without correcting upstream stressors [57,59,60,61].

(**8**) ER stress. Protein overload ER stress is *upstream and mechanistically distinct* from the ISR. It reflects protein folding burden, secretory load, and ER-associated degradation (ERAD) capacity, which are especially critical in trophoblast and primitive endoderm lineages with high secretory demands. ER stress can exist without full ISR engagement, and conversely ISR can be triggered by non-ER stressors. Separating this category allows discrimination between embryos failing due to proteostasis bottlenecks vs. those failing due to *global translational repression*. This distinction is essential for mechanism-based mitigation (e.g., chaperone support vs. ISR modulation) [25,62,63,64].

(**9**) Mitochondrial/oxidative stress. This category captures *mitochondrial adaptive remodeling, *not ROS per se. It includes mitochondrial translation, fission–fusion dynamics, and the mitochondrial unfolded protein response (UPR). These processes report whether mitochondria are repairing ETC components, adjusting morphology, and restoring ATP capacity. Importantly, mitochondrial stress responses can be activated* without overt ROS escalation, *representing a compensatory phase between initial stress sensing (category 3) and oxidative damage (category 10). Thus, this group identifies embryos still capable of recovery through mitochondrial quality control [65,66,67,68,69].

(**10**) ROS escalation. Here ROS are no longer signaling intermediates but *uncontained oxidants causing damage. *This category marks the transition from adaptive redox signaling to genotoxic stress, lipid peroxidation, SAPK activation, and apoptosis. Conceptually, it represents *failure of antioxidant systems, mitophagy, and metabolic rewiring *identified in categories 3 and 9. ROS escalation may strongly predict irreversible developmental loss and hypothetically aligns temporally with blockage of DASR commitment rather than ISR resolution [70,71,72,73].

(**11**) Aerobic glycolysis–oxidative phosphorylation switching. This category reflects the *developmental metabolic decision downstream of stress signaling.* Early embryos and stem cells favor aerobic glycolysis to support rapid biosynthesis. Strong or critical stress suppresses glycolysis and forces a shift toward oxidative phosphorylation to maximize ATP per carbon, consistent with the Cantley principle [74,75,76,77]. While adaptive short-term, this shift limits anabolic growth and correlates with slowed proliferation, altered lineage allocation, and may lead to implantation failure. Thus, this category reports *functional metabolic consequences*, not stress sensing itself, and explains why metabolic signatures integrate multiple upstream stress axes.

Stress adaptation programs enable limited developmental success during early embryogenesis. These programs are constrained and failure prone. While multiple factors—including chromosomal abnormalities, uterine environment, and stochastic developmental variation—are known to influence early pregnancy loss, accumulating evidence from stem cell models, embryo transcriptomic analyses, and embryoid systems suggests that failure to resolve the Integrated Stress Response (ISR) and transition to adaptive developmental programs may represent a mechanistic contributor in a subset of cases.

In this context, we propose that stress resolution dynamics, particularly the transition from ISR to downstream developmental programs, provide a useful framework for interpreting how diverse perturbations may converge on common developmental outcomes. This framework is intended to complement, rather than replace, existing models of early embryo failure.

## 7. Comparing Direct Stress GO Group Gene Marker Testing of Stressed Human Embryos vs. Stem Cell Type Dose-, Time- and Qualitatively-Stressed Markers Previously Filtered by Testing These Against the Stress GO Group Gene Markers

This approach compares direct stress GO group markers measured in stressed human embryos with dose-, time-, and qualitatively-defined stress markers previously established in stem cell models and validated against these same GO-based stress signatures. As described by Chousal et al. [20], bulk RNA-seq of entire embryos classified as good morphology (GM) or poor morphology (PM) provides a spatially informed analysis when embryo halves are isolated prior to sequencing, rather than focusing on cell-type heterogeneity. This approach enables quantitative recovery of all cells and transcripts within each half, increasing confidence in transcriptomic comparisons within single embryos. In turn, this strengthens comparisons between embryos, including analyses of pole-to-pole differences and IVF-associated stochastic variation [20].

Bulk RNAseq enables deeper transcript read depth by starting with orders of magnitude more input mRNA per embryo. This also enhances confidence in single embryo inter-polar comparisons and enables better detection and quantitation of important low abundance genes such as stress response and lineage-determining transcription factors and ligand receptor function in stressed developmental response. Higher read counts also supports more stable expression estimates, lower technical zero count inflation, and lower amplification bias, which is necessitated, in contrast, by scRNAseq use of higher IVT prior to library creation and high stochastic noise from early amplification steps of low abundance and/or high GC mRNA fuels uncertainty of important low copy number genes. Higher copy number bulk RNAseq obviates many problems caused by scRNAseq methods.

In Chousal et al. [20], deconvolution is used to emulate some parts of the analysis supported directly by scRNAseq. This enabled one line of evidence for the relative numbers of cells in the trophoblast embryonic and yolk sac endoderm lineages in PM vs. GM embryos and supported the conclusion that 1st differentiation ESC lineage PrimEndo is the key deficiency associated with implantation failure. Some ligand–receptor interactions were supported by CellPhoneDB [78,79], but the Scenic software for transcription factor function [80] across lineages was not used.

2024–2025 has been an annus mirabilis year of studies on the importance of primitive endoderm. Diverse studies from diverse models from five labs can be integrated into a whole hypothetical model for primitive endoderm in early mammalian development (Supplemental Table S2).

Recent studies across multiple experimental systems collectively highlight the importance of primitive endoderm (PrE) but also reveal important differences in how PrE function and dysfunction are defined.

In human embryo datasets, Heidi Cook-Andersen and colleagues identify [20], PrE deficiency as a correlate of implantation failure in poor morphology embryos, based on bulk RNA-seq and deconvolution approaches. In contrast, stem-cell-based systems interrogate PrE potential under controlled perturbations. For example, RACL culture conditions induce an intermediate state between naïve pluripotency and PrE, suggesting expanded developmental plasticity under specific signaling environments with required LIF receptor activity to maintain an intermediate totipotent state between naïve ESCs and primitive endoderm, a sort of universal cellular repair kit [81]. In mouse ESC stress models, strong stressors induce dose-dependent override of naïve pluripotency toward PrE lineages [1], suppressing formative pluripotency compared with ND. Interestingly, one cluster of the highest dose of strong stress in the scRNAseq analysis showed strong LIF receptor signaling through Stat3, Klf4 and Tbx3, similar to the LIF receptor hyper-signaling totipotent cells obtained with RACL media [81]. Unlike strong stressors, qualitatively weaker stressors preserve lineage balance, similar to ND, while suppressing stemness [5]. These studies produced dose-dependent strong and qualitatively strong or weak transcriptomic markers. In embryoid systems, forced expression of GATA6 enhances PrE representation and improves blastocyst-like progression efficiency from the previous 10% per day to 80% per day [21], and this improved developmental progression of the embryoids to test the effects of the potentially toxic effects of nicotine, caffeine, and alcohol. Possible synergistic integration of these diverse studies can be accomplished by using the strong vs. weak and dose-dependent strong markers to characterize stress states across markers in all of these models.

Taken together, these studies differ in key dimensions: (i) model system (human embryos vs. mouse ESCs vs. embryoids), (ii) perturbation type (endogenous embryo variability, culture conditions, stress exposure, or genetic manipulation), and (iii) readout (correlation with implantation failure vs. induction of lineage states vs. functional rescue). While they converge on the importance of PrE in supporting early developmental competence, they do not yet define a single unified mechanism. Notably, most studies have not explicitly interrogated stress as a unifying upstream driver of PrE deficiency or misallocation.

However, in Chousal et al. [20], there were studies of how poor morphology embryos markers associated with the likelihood of implantation failure but they did not study stress, which is often associated with embryo loss or deficiency. One method to test for stress affects associated with implantation failure would be to directly test GO groups for functional analysis of PM or GM embryos of the *11 key stress types* above and stem cell time-, dose-, and quality-dependent markers for insight into lineage effects contributing to PM outcomes (Table 1).

## 8. Direct Screening of Stress GO Group Genes into Embryos Would Be Used in This Method

*Workflow**2B:* The direct use of GO groups for different stress types is generally useful for pinpointing types of stress in either pole of a PM human IVF embryo and correlating that with primitive endoderm failure and the two insufficiencies defining implantation failure and miscarriage: (1) absence of hCG and (2) probable failure to adhere and invade endometrial stroma and connect to maternal blood during the lacunar stage of implantation.

*Workflow**3:* However, by pre-filtering these GO group genes through time- and dose-dependent ESC and TSC and strong vs. weak qualitative markers first, the cell type specific DEGs can pinpoint stresses through specific cells in each pole with implantation failure and thus define defects in primitive endoderm and hCG and adhesion failure [82] in the TSC lineage.

For time-dependence resolution of ISR markers, early post stress responses tend to be cell autonomous and with low total DEGs, which are highly downregulated (70–80%), whereas after ISR and stress adaptation, or in ND, longer-term resolved stresses tend to have total DEGs at higher levels and with much greater percent upregulated (40–60%).

*Workflow 4:* Use custom sets of genes from different parent and child GO groups in the same stress category that corroborate pathway activation and pathway transcriptomic outcomes. These assembled GO groups simultaneously confirm pathway activity levels and type of dependent transcription addressing ISR and DASR outcomes dependent on each pathway. This may help pinpoint possible mechanisms and methods of mitigation.

*Master Table of GO groups for the 11 types of stress and for categories of function validation in each category. *The rationale and details of these 11 categories of GO groups are discussed above and applied here to test for stressed-stem-cell-specific category usage and to apply these also directly to human embryos, isolated stem cells (i.e., cervical TRIC trophoblasts, and huIPSC-huESC) to assess stem-cell-specific stress category usage and outcome.

## 9. ORA–FEA vs. GSEA: Complementary Roles in Developmental Stress Analysis

ORA–FEA and GSEA provide complementary approaches to enrichment analysis. ORA–FEA operates on discrete sets of differentially expressed genes (DEGs) and is well-suited for identifying statistically significant, high-confidence pathway activation associated with defined biological thresholds, such as NOAEL, LOAEL, or IC50 [83,84]. This makes it particularly useful in contexts where discrete biological decision points are of interest, such as ISR resolution or lineage-specific failure.

GSEA evaluates enrichment across ranked gene lists and detects distributed transcriptional changes [85,86]. This makes GSEA especially valuable for detecting early or subtle stress responses, as well as coordinated pathway shifts across gene sets. It is also extremely important in studies of embryo markers and for developmental pathways where transcript numbers may be low and can mediate important events at low numbers [87,88].

Rather than viewing these methods as competing, we use them in a complementary manner: ORA–FEA to define high-confidence functional decision points and GSEA to extend sensitivity across broader transcriptomic trends [83,86]. Together, these approaches provide both specificity and sensitivity in characterizing developmental stress responses. A comparison of transcriptomic analysis methods is given for broad criteria of comparison of analyses (Table 2).

ORA–FEA’s strengths are its ease to interpret. Here is a simple explanation, and detailed reviews should be understood as well to complete preparation for stress analysis [83]. ORA–FEA works well with biologically curated DEG lists. It aligns with toxicology thresholds and discrete outcomes and provides an immediate view of the relative significance of different stress GO groups in stressed TSC or test PM embryos marker sets and the functions for each group. For dose-, time-, and quality-dependent stress marker sets, trends in significance across the variables enables most important functions in stem cells of stressed embryos. Thus, it is important to pinpoint functional emphasis of stressed stem cells, embryos, or embryoids as they adapt to stress across the range of magnitude of these variables.

GSEA’s strengths are that it is sensitive to subtle, distributed shifts in marker and function emphasis across these ranges of magnitude. GSEA does not require hard DEG cutoffs and is thus useful for early or low-signal stress functions. This is a simple explanation, and detailed reviews should be understood as well [86]. GSEA analysis of stressed ESC data enables analysis of 10–20-fold more DEGs than ORA–FEA for recent dose- [17] and quality-dependent [5] transcriptomes.

ORA–FEA is important here since the stress biology compared at biologically equivalent exposures depends on decision points (e.g., ISR resolution, lineage loss) and the significance of these functions in leading to decisions. Thus, DEG-based thresholds match biological equivalence logic. ORA–FEA is a foundation and GSEA is complementary, not foundational. This is a simple comparison of the two techniques for Analysis of Enrichment, and a detailed review should be understood as well [89].

## 10. Part 3—Statistical Analysis of Stressed-Stem-Cell and Stressed-PM-Embryo Markers Should Enable Ranking to Predict PM Embryos Most Likely to Produce Live Birth and GM Embryos Most Likely to Produce Implantation Failure

***A first analytical output.*** This is typically a stress TSC or ESC DESM spreadsheet used for a hypergeometric analysis carried out to create an ORA–FEA of significance of functional homeostatic responses. These are filtered in steps through a second analysis.

***A second analysis*** will use GSEA stats (rank order of all DEGs whether significant or not, ranking can be by significance, fold change, or both) for a more nuanced analysis of low copy number DEG events of high biological significance.

***A third analytical output*** is functions used by different cells at different doses or durations of stress. This is a functional analysis (filter) to understand what functions are needed early or at low dose and persist into later DEGs and higher dose DEGs because they are still needed (not solved completely in the homeostatic response). And unique functions that arise for stem cells to adapt to higher doses or longer durations of stress are a second statistically defined group of high biological significance.

## 11. Part 4—Statistical Analysis of Pooled Single PM Embryos to Address Ranking of PM Embryos for Most Likely to Produce Live Birth and GM Embryos Most Likely to Produce Implantation Failure

**Stress biology** → **workflow.** Having established that stress outcomes depend on dose, time, and qualitative severity, we next describe how these dimensions are experimentally resolved and translated into transcriptomic benchmarks.

**GO categories → stem-cell filtering.** While GO categories define stress functions broadly, their developmental relevance emerges only when filtered through lineage-resolved stem cell stress responses.

**Stem cells → embryos.** These stem-cell-resolved stress programs provide the necessary reference space to interpret bulk embryo transcriptomes, where lineage signals are otherwise obscured by averaging.

## 12. Ratio-Based Analysis of Embryonic–Abembryonic (EM–AB) Pole, and Counterpoised Pathway Asymmetry

To quantify spatially polarized transcriptional programs within pooled or individual embryos, we propose to analyze **single-embryo RNA-sequencing data** by contrasting gene-level and gene-set-level signals between the **embryonic (EM)** and **abembryonic (AB)** poles or between counterpoised pathways (i.e., stemness vs. differentiation or aerobic glycolysis vs. ox-phos metabolism. For each embryo, DEGs and ORA–FEA should be computed independently for each pole and counterpoised functional GO groups, followed by **ratio- or difference-based comparisons** between counterpoised pathways or embryonic poles.

This strategy builds on the previous use of **counterpoised biological programs** to infer cellular and developmental state, including stemness vs. differentiation, proliferation vs. quiescence, and aerobic glycolysis vs. oxidative phosphorylation [86,90,91]. In early embryo and stem cell systems, such contrasts have proven especially powerful because developmental competence is often encoded not in absolute expression levels but in **relative balance between competing transcriptional programs**.

*Gene-set scoring and construction of stress- and development-resolved indices.* For each embryo and pole, pathway-level activity was summarized using ORA–FEA and conceptually related **single-sample gene-set scoring frameworks**, including Gene Set Variation Analysis (GSVA), module scoring, and AUCell-based approaches [80,90,92,93]. These methods provide per-sample or per-cell estimates of coordinated gene-set activity and are widely used in high-profile stem cell and developmental transcriptomic studies.

## 13. Finally, We Introduce Quantitative Indices—Including a Stress Dose–Response Index (SDRI), Stress Time–Resolution Index (STRI), and Stress Qualitative Response Index (SQRI) for Quantitating Stress in Clinical Samples

These are used to assess methods to rank embryos by likelihood of implantation success or failure at both cohort and single-embryo resolution. Together, this framework provides a biologically grounded, lineage-aware method to interpret embryo transcriptomes, identify stress mechanisms underlying poor morphology and implantation failure, and guide embryo selection, culture optimization, and experimental embryoid design.

The SDRI, SQRI, and STRI indices presented here are not yet clinically validated predictive tools but rather represent a hypothesis-generating framework grounded in experimentally defined stem cell stress–response programs and their integration with embryo transcriptomic data. While these indices are designed to enable quantitative ranking of stress states and developmental trajectories, their predictive performance for clinical outcomes such as implantation, chemical pregnancy loss, and live birth remains to be established.

Rigorous validation will require prospective studies integrating trophectoderm biopsy transcriptomics with known implantation and pregnancy outcomes, enabling quantitative evaluation using receiver operating characteristic (ROC) curves and related performance metrics. Such studies would determine the sensitivity, specificity, and predictive value of these indices and assess their utility relative to existing embryo selection methods.

Pole-specific scores should then be contrasted (e.g., EM minus AB, or log_2_[EM/AB]) to generate **biologically interpretable indices,** summarizing three orthogonal properties of stress-responsive developmental regulation that have been defined and validated through experimental stem cell models in our laboratory:

1. **Stress Dose–Response Index (SDRI)**: Quantifies the magnitude of transcriptional activation across curated stress-responsive gene sets derived from graded stress exposures in ESCs and TSCs [17]. *“How strong is stress activation right now?”*
2. **Stress Quality Response Index (SQRI)**: Distinguishes adaptive (“weak”) vs. maladaptive (“strong”) stress programs based on qualitative differences in gene-set usage, originally defined using ESC transcriptomic responses and subsequently extended to trophoblast and embryo contexts [5] *“Is this strong enough to cause compensatory and prioritized differentiation to 1st lineage, or weak, which overrides stemness but emulates ND?”*
3. **Stress Time–Resolution Index (STRI)**: Positions embryos along a temporal trajectory from the immediate Integrated Stress Response (ISR) Toward Delayed Adaptive or Developmental Stress Responses (DASRs), based on re-analysis of embryo time-course datasets [94] and extended in a recent systems-level re-analysis identifying an ISR→DASR transition (unpublished data). *“How far along the hypothetical ISR→DASR resolution trajectory is this sample?”*

These indices represent summaries of counterpoised transcriptional programs, analogous to previously published indices derived from single-sample enrichment scores in cancer biology, metabolism, and differentiation systems [91,95]. A single-sample transcriptomic marker set scoring will not be discussed here. However, single-sample gene-set scoring methods include GSVA, ssGSEA, SLEA/z-score approaches, PLAGE, singscore, AUCell/UCell, and newer ensemble or benchmarked methods [96]. The best choice depends on sample number, batch effects, whether the sample is bulk or single-cell, and whether the goal is pathway interpretation or clinical classification. Importantly, while the statistical framework draws on established bioinformatics methodology, the biological definition of the gene sets and the interpretation of the indices are grounded in experimentally validated ESC and TSC stress-response models developed by the Rappolee laboratory and related groups.

By applying these indices at the level of individual embryos and across EM–AB poles or between counterpoised programs, this framework may enable quantitative ranking of embryos by predicted developmental competence and provides a mechanistic bridge between stem cell stress models and early human embryo outcomes.

## 14. Standard ORA–FEA (Baseline)

For a given background gene universe of size *U*, containing *K* genes annotated to a specific GO term or curated gene set, and a DEG list of size *n*, the probability of observing *k* or more overlapping genes by chance is calculated as:

p=∑i=kmin(K,n)(Ki)(U−Kn−i)(Un)

where

- *U* = total number of genes eligible for testing (the background universe);
- *K* = number of genes in the GO term or gene set;
- *n* = number of DEGs in the query list;
- *k* = number of genes shared between the DEG list and the gene set.

In plain terms, ORA–FEA asks:

*If we randomly selected n genes from all U testable genes, how likely would we be to observe k **or more** genes belonging to a particular functional category purely by chance?*

The importance of carefully defining the background gene universe (**U**) has been emphasized repeatedly, as inappropriate universe selection can substantially bias enrichment statistics [97,98]. Despite its simplicity, ORA–FEA remains a robust baseline approach when DEGs are well-defined and has been extensively applied in studies of stem cell identity, early embryonic development, and placental biology, including analyses of pluripotency maintenance, lineage commitment, and stress-induced transcriptional remodeling [86,99,100].

In a recent study, ORA–FEA is used as a baseline functional statistic against which more structured, lineage-aware, and stress-resolved indices are compared [unpublished data]. This approach allows direct integration of classical enrichment statistics with experimentally defined stress-response gene sets derived from stem cell and embryo models developed by our laboratory and others.

## 15. Gene Set Enrichment Analysis (GSEA): Rank-Based Functional Enrichment

In contrast to ORA–FEA, which tests enrichment within a discrete list of significant DEG, Gene Set Enrichment Analysis (GSEA) evaluates whether genes from a predefined set are non-randomly distributed across a ranked list of all expressed genes (e.g., ranked by log_2_ fold change or other continuous metrics). This avoids reliance on arbitrary DEG thresholds, greatly increasing sizes of marker sets, and enables detection of coordinated but modest expression shifts across many genes.

### GSEA Enrichment Score (ES)—Explicit Subramanian Formulation [86]—Where the Running-Sum Components Are

$$P_{hit}\left(S,i\right)=\sum_{g_{i}\in S,\;\;j\leq i\;}{\left|r_{j}\right|}^{p}\;/\;N_{R},\;where\;N_{R}=\sum_{g_{i}\in S}{\left|r_{j}\right|}^{p}$$

$$P_{miss}\left(S,i\right)=\sum_{g_{i}\notin S,j\leq i}1\;/\;{(N-N}_{H})$$

where

- N = total number of genes in ranked list;
- S = gene set;
- ∣S∣ = number of genes in the set;
- $r_{j}$ = ranking metric (e.g., log_2_FC, signal-to-noise);
- p = weighting exponent (usually p=1);
- j = position in ranked list;
- i = running index through ranked list.

The enrichment score (ES) is calculated as the maximum deviation of a weighted running-sum statistic across the ranked gene list, where $P_{hit}(j)$ and $P_{miss}(j)$ represent the weighted contributions of genes inside and outside the gene set, respectively, and depend on the ranking metric $r_{j}$, gene set size ∣S∣, and total gene number N.

In plain terms, GSEA asks whether genes belonging to a pathway are preferentially located near the top or bottom of the ranked transcriptome, for log2FC, rather than randomly distributed.

GSEA complements ORA–FEA by increasing statistical sensitivity and effective sample size through inclusion of all expressed genes, enabling detection of early, low-amplitude, or distributed stress responses that may not meet DEG thresholds. In this framework, ORA–FEA identifies discrete, biologically meaningful decision points in stress adaptation (e.g., ISR resolution or lineage imbalance), while GSEA refines these findings by revealing broader transcriptomic trends across dose-, time-, and quality-dependent stress conditions.

## 16. Summary

This review has outlined a two-step methodology to provide stem cell-specific stress markers and use them to analyzed time- dose- and quality-dependent stress responses and apply these markers to a clinical IVF embryo sample, using markers from 11 categories of stress responses provided by GO groups that report presence and upregulation, including activity and adverse effect outcomes.

## Figures and Tables

**Table 1:** Diverse models of early mammalian development suggest that primitive endoderm function integrates into several normal and stressed outcomes.

Citation	Model	Perturbation	PrE Finding	Interpretation
Chousal et al., 2024 [20]	Human IVF embryos	PM vs. GM quality AB vs. EM pole	In PM embryos, primary PrE insufficiency	Correlates with implantation failure and miscarriage, transcriptomic markers
Linneberg- Agerholm et al., 2024 [81]	huESC mouse embryo	RACL media	PrE-ICM naïve endoderm, totipotent Hyper-LIF, R signal-dependent	Expanded plasticity, universal cell toolkit (?)
Jorgensen, et al., 2025 [21]	mouse embryoid	(1) ESC: Gata4 PrE (2) ESC, (3) TSC	PrE from Gata4 ESC enables high development, progress	Increased PrE improves efficiency, stress tests
Ruden and Singh, et al. 2024 [17]	Rex1RFP ESC mouse	Hyperosmotic strong stress doses	Dose-dependent PrE lineages up, intermediate PrE-Naïve ESC is Hyper LIF, R DEG up	Transcriptomic PrE, PE, and VE markers up, formative down
Singh and Ruden, et al. 2025 [5]	FUCCI ESC mouse	Weak, strong stress categories	Strong stress–PrE up, formative ESC down, weak stress or ND induces both lineages	Dose-dependent strong marker, quality-dependent, strong vs. weak transcriptomic markers identified

**Table 2:** Transcriptomic analysis methods, strengths, limitations and best uses.

Method	Strength(s)	Limitation(s)	Best Use Case
ORA–FEA	High specificity, interpretable	Requires DEG threshold	Decision points, strong effects
GSEA	High sensitivity, no threshold for DEG	Less intuitive interpretation	Subtle/early responses
Bulk RNA-seq	Deep coverage important for rate-limiting, lineage-determining genes	Loses cell-type resolution	Whole-embryo or pole-level analysis, stable quantitation
scRNA-seq	Cell-type resolution	Dropout, noise, lower depth	Lineage identification
Spatial	Spatial context	Lower gene depth	Tissue architecture transcriptomics
Multi-omics	Integrative biology	Complexity, cost	Mechanistic inference

## Data Availability

The data presented in this study are GO group marker training sets to define ISR and DASR functions are available in MGI at **https://www.informatics.jax.org/** (accessed on 21 May 2026).

## Supplementary Materials

The following supporting information can be downloaded at: https://www.mdpi.com/article/10.3390/cells15110996/s1, [Supplemental Table S1: Core (7) and Expanded (10) Stress Sensor–Effector Sentinel Pathways. Supplemental Table S2: Stress-Responsive GO, GOBP, and Hallmark Gene Sets Used to Define risks of Adverse Stem Cell and Preimplantation Embryo Outcomes. Supplemental Table S3: Expanded Gene Sets to Distinguish PrE vs VE vs PE (Mouse-focused), and Supplemental Table S4: Expanded Gene Sets to Distinguish TSC lineages (Mouse-focused). References [101,102,103,104,105,106,107,108] are cited in the Supplementary Materials.

## Abbreviations

**AB**	abembryonic pole of the blastocyst
**AMPK**	AMP-activated Protein Kinase; a cellular energy sensor regulating metabolism and growth under stress
**ARE**	Antioxidant Response Element; cis-regulatory element activated during oxidative stress (e.g., NRF2 signaling)
**BaP**	benzo[a]pyrene; a polycyclic aromatic hydrocarbon mutagen
**CEPT**	Chroman 1, Emricasan, Polyamines, trans-ISRIB; a small-molecule cocktail including a PK inhibitor and ISR modulator used to improve embryoid development.
**DASR**	Developmental Adverse Stress Response; maladaptive stress state associated with developmental failure
**DEG**	Differentially Expressed Gene
**EM**	Embryonic pole of the blastocyst
**ER**	Endoplasmic reticulum
**ESC**	Embryonic Stem Cell
**FDR**	false discovery rate; adjusted *p*-value for multiple testing
**GM**	good morphology embryo
**GO**	Gene Ontology
**GOBP**	Gene Ontology Biological Process
**GR**	glucocorticoid receptor (NR3C1)
**GSEA**	Gene Set Enrichment Analysis; ranking-based pathway analysis
**hCG**	Human Chorionic Gonadotropin
**HTS**	high-throughput screening
**IC50**	inhibitory concentration 50%; dose causing 50% growth inhibition
**ISR**	Integrated Stress Response
**IVF**	In Vitro Fertilization
**KEGG**	Kyoto Encyclopedia of Genes and Genomes
**LOAEL**	lowest observed adverse effect level
**MAPK**	Mitogen-Activated Protein Kinase
**MGI**	Mouse Genome Informatics
**MSigDB**	Molecular Signatures Database
**ND**	normal differentiation (without stress)
**NOAEL**	no observed adverse effect level
	Over-Representation Analysis–Functional Enrichment Analysis
**PERK**	Protein kinase RNA-like endoplasmic reticulum kinase (EIF2AK3)
**PK**	protein kinase
**PM**	poor morphology embryo
**ROS**	Reactive Oxygen Species
**SAPK**	Stress-Activated Protein Kinase (e.g., JNK/p38).
**scRNAseq**	Single-cell RNA sequencing
**SDRI**	Stress Dose–Response Index *(author-defined metric)*
**SG**	stress granule
**SQRI**	Stress Qualitative Response Index *(author-defined metric)*
**STRI**	Stress Time–Resolution Index *(author-defined metric)*
**TSC**	Trophoblast Stem Cell
**UPR**	Unfolded Protein Response
