# Supplementary material for: Systematic Methods to Resolve Lineage-Specific Stress States in Early Mammalian Embryos and That May Enable Miscarriage Prediction
Source: Cells. 2026 May 28;15(11):996. doi: 10.3390/cells15110996 (PMC13256741; doi:10.3390/cells15110996)
Supplement: Supplementary file 1 [file cells-15-00996-s001.zip › cells-4273843 0513 Supplemental Table S1. Core (7) and Expanded (10) Stress Sensor¿CEffector Sentinel Pathways from 21st century vision 4 30 2026.pdf]

Supplemental Table S1. Core (7) and Expanded (10) Stress Sensor–Effector Sentinel Pathways. These were suggested and expanded by the report from the *Committee on Toxicity Testing and Assessment of Environmental Agents. Toxicity testing in the 21st century : A vision and a strategy*

#	Toxicological Domain	Primary Sensor Mechanism	Effector Transcription Factor	DNA Response Element	Major Biological Program
1	Oxidative / Electrophile Stress	KEAP1 cysteine redox sensing	NRF2 (NFE2L2)	ARE	Antioxidant and detoxification genes
2	Proteotoxic / Heat Shock	Misfolded protein accumulation; chaperone titration	HSF1	HSE	Heat shock proteins; proteostasis
3	ER Stress / UPR (ATF6 arm)	ER protein misfolding	ATF6	ERSE	ER chaperones; ERAD
4	DNA Damage / Genotoxic Stress	ATM/ATR kinase signaling	p53	p53 RE	Cell cycle arrest; DNA repair
5	Inflammatory / Innate Stress	IKK activation	NF-κB	NF-κB RE	Cytokines; inflammatory mediators
6	Stress Kinase / MAPK	JNK/p38 activation	AP-1 (JUN/FOS)	AP-1 RE	Adaptive stress transcription
7	Heavy Metal Stress	Intracellular metal accumulation	MTF1	MRE	Metallothionein's; metal detoxification
8	Hypoxia	Oxygen-dependent prolyl hydroxylase regulation of HIF1α	HIF1α	HRE	Angiogenesis; glycolysis; hypoxic adaptation
9	Xenobiotic Receptor AhR |	Direct ligand binding	AhR (ARNT dimer	XRE	Phase I metabolism (dioxins, PAHs, planar Aromatics (CYP1A1 etc.)
10	Hyperosmotic Stress	Increased intracellular	NFAT5 (TonEBP)	TonE	Osmo-protective ionic strength genes; cellular osmoregulation

[1]
[2-4]

1. Simmons, S.O.; Fan, C.Y.; Ramabhadran, R. Cellular stress response pathway system as a sentinel ensemble in toxicological screening. *Toxicol Sci* **2009**, *111*, 202-225.
2. Andersen, M.E.; Krewski, D. The vision of toxicity testing in the 21st century: Moving from discussion to action. *Toxicol Sci* **2010**, *117*, 17-24.
3. Andersen, M.E.; Krewski, D. Toxicity testing in the 21st century: Bringing the vision to life. *Toxicol Sci* **2009**, *107*, 324-330.
4. National Research Council (U.S.). Committee on Toxicity Testing and Assessment of Environmental Agents. *Toxicity testing in the 21st century : A vision and a strategy*. National Academies Press: Washington, DC, 2007; p xvii, 196 p.
