# Supplementary material for: Systematic Methods to Resolve Lineage-Specific Stress States in Early Mammalian Embryos and That May Enable Miscarriage Prediction
Source: Cells. 2026 May 28;15(11):996. doi: 10.3390/cells15110996 (PMC13256741; doi:10.3390/cells15110996)
Supplement: Supplementary file 1 [file cells-15-00996-s001.zip › cells-4273843 0513 Supplemental Table S2 update 5 12 2026.pdf]

Supplemental Table S2. Stress-Responsive GO, GOBP, and Hallmark Gene Sets Used to Define risks of Adverse Stem Cell and Preimplantation Embryo Outcomes.

Axis	Pathway State	GO / Gene Set	Source	What This Reveals
**1 Pan-Stress Protein Kinases (AMPK vs SAPK)**				
**What this reveals: Discriminates adaptive metabolic stress (AMPK-dominant) from inflammatory, oxidative, or genotoxic stress (SAPK/JNK/p38-dominant). This axis predicts whether embryos enter reversible quiescence or irreversible arrest/senescence.**				
**Axis**	**Pathway State**	**GO / Gene Set**	**Source**	**What This Reveals**
Pan-Stress PK	Pathway present / active	stress-activated protein kinase signaling cascade (**GO:0031098**) **16 genes**	GO	Core JNK/p38 stress signaling activated by diverse stressors
Pan-Stress PK	Activation tuning	regulation of stress-activated MAPK cascade (**GO:0032872**) **39 genes, 71 annotations**	GO	Magnitude, persistence, and recurrence of stress
Pan-Stress PK	Outcome / sequelae	inflammasome-mediated signaling pathway **(GO:0141084**) **77 genes, 161 annotations**	GO	Commitment to maladaptive or chronic stress
Pan-Stress PK	Pathway present	AMP-activated protein kinase signaling pathway (**GO:0031929**)	GO	Energy stress sensing and metabolic adaptation
Pan-Stress PK	Outcome	autophagy of mitochondrion**; (GO:0000422**), **109 genes, 228 annotations**	GO	Adaptive survival versus exhaustion
**2. Stress Granules (Assembly ↔ Disassembly)**				
What this reveals (thumbnail): Stress granules act as a reversible buffer between acute ISR and long-term DASR. Disassembly is the critical indicator of stress resolution capacity and return to developmental progression.				
**Axis**	**Pathway State**	**GO / Gene Set**	**Source**	**What This Reveals**
Stress Granules	Assembly	stress granule assembly (**GO:0034063**), **44 genes, 92 annotations** (Parent, should follow children if interesting)	GO	Entry into acute translational arrest
Stress Granules	Structure	Mouse stress granule**, 37 genes** The proteins and RNA aggregation, arrangement, and bonding of molecules; forming stress granule **(GO:0062028), 10 genes,** .	MSigDB **MM6875**	Cytoplasmic sequestration of stalled ribosomes
Stress Granules	Resolution	**22 annotations**	GO	Capacity to resolve ISR and resume development
Stress Granules	Disassembly	disaggregation of a stress granule to its constituent protein and RNA parts **(GO:0035617); 7 genes**	**M23365**	Coordinated stress-granule biology
Stress Granules	Outcome	negative regulation of translational initiation **(GO:0045947**) 46 **genes**, 69 **annotations**	GO	Precedes SG assembly initiation and is causal for it
**3 ROS / Oxidative Stress (Mitochondrial Emphasis)**				
What this reveals: Mitochondria are early integrators of stress in embryos. This axis determines whether ROS is contained through adaptive responses or escalates into DNA damage, SAPK activation, and apoptosis.				
**Axis**	**Pathway State**	**GO / Gene Set**	**Source**	**What This Reveals**
ROS Stress	Stress present	cellular response to ROS (**GO:0034614**) **164 genes, 293 annotations**	GO	Cellular exposure to reactive oxygen species
ROS Stress	Genotoxic	intrinsic apoptotic signaling pathway in response to DNA damage (**GO:0008630**) **115 genes, 244 annotations**	GO	Transition to genome instability
ROS Stress	Mito function	inner mitochondrial membrane protein complex (**GO:0098800**), **43 genes, 124 annotations**	GO	Bioenergetic competence

ROS Stress	Outcome	apoptotic mitochondrial changes, (**GO:0008637**) **126 genes, 314 annotations**	GO	Irreversible failure
ROS Stress	Recovery	mitophagy (**GO:0000422**) **109 genes, 228 annotations**	GO	Adaptive mitochondrial cleanup

4 Hormonal Stress (Cortisol / GR)

What this reveals: A ligand-dependent, receptor-mediated stress axis that must be distinguished from generic stress. Indicates maternal or environmental hormonal programming of embryo developmental trajectory.

Axis	Pathway State	GO / Gene Set	Source	What This Reveals
Hormonal Stress (GR)	Capacity	nuclear glucocorticoid receptor activity (**GO:0004883**), **1 genes, 2 annotations; NR3C1**	GO	Ability to respond to glucocorticoids
Hormonal Stress (GR)	Pathway active	response to cortisol **(GO:0051414) 5 genes, 6 annotations**<br>response to corticosterone **(GO:0051412) 23 genes, 24 annotations**	GO	Direct cortisol signaling
Hormonal Stress (GR)	Pathway active	response to glucocorticoid **(GO:0051384) 158 genes, 222 annotations**	GO	Canonical glucocorticoid receptor output
Hormonal Stress (GR)	Direct targets	**GR_Q6 motif set, 222 genes** NR3C1 binding sites direct promoter response	MSigDB	Direct NR3C1 binding
Hormonal Stress (GR)	Outcome	GOBP_RESPONSE_TO_CORTISOL **, 9 genes**	GO **M23990.**	Endocrine-driven metabolic reprogramming

5. Aneuploidy / Chromosomal Imbalance

What this reveals: Indicates chromosome mis-segregation, gene dosage imbalance, and mitotic checkpoint activation—among the strongest predictors of preimplantation developmental arrest and implantation failure.

Axis	Pathway State	GO / Gene Set	Source	What This Reveals
Aneuploidy Stress	Presence	positive regulation of chromosome segregation (**GO:0051984**) **30 genes, 65 annotations**<br>G2/M transition of mitotic cell cycle<br>(**GO:0000086**) **146 genes, 364 annotation**	GO	Mitotic fidelity
Aneuploidy Stress	Checkpoint	Mitotic spindle assembly checkpoint signaling (**GO:0007094**)<br>**51 genes, 131 annotations**	GO	Mitotic arrest
Aneuploidy Stress	Surveillance	DNA damage checkpoint (**GO:0000077**) **124 genes, 300 annotations**	GO	Genome surveillance
Aneuploidy Stress	Hallmark	**HALLMARK_MITOTIC_SPINDLE 199 genes**	MSigDB	Chromosomal instability
Aneuploidy Stress	Outcome	mitotic cell cycle checkpoint signaling (**GO:0007093**), **148 genes, 300 annotations**	GO	Developmental block

6. Mutagenic / Genotoxic Stress

What this reveals: Tracks endogenous or exogenous DNA damage arising from replication stress, oxidative injury, or environmental mutagens.

Axis	Pathway State	GO / Gene Set	Source	What This Reveals
Genotoxic Stress	Damage present	nucleotide-excision repair (**GO:0006289**) **78 genes, 181 annotations**	GO	Presence of DNA lesions
Genotoxic Stress	Repair	*MMR-mismatch repair*<br>(**GO:0006298**) **26 genes, 75 annotations.**<br>DSB -HR *double-strand break repair via homologous recombination.*<br>**GO:0000724 186 genes, 516 annotations.**<br>DSB-NHEJ *double-strand break repair via nonhomologous end joining.*<br>**GO:0006303 77 genes, 214 annotations.**<br>*DNA alkylation repair*<br>**GO:0006307 9 genes, 25 annotations.**	GO	Repair engagement
Genotoxic Stress	Checkpoint	**HALLMARK_P53_PATHWAY**<br>p53 signaling pathway, mouse, **200 genes**	MSigDB **MM3896**	Damage sensing and fate control

Genotoxic Stress	Hallmark	**HALLMARK_DNA_REPAIR mouse, 148 genes**	MSigDB	Coordinated repair program
Genotoxic Stress	Outcome	cellular senescence (GO:0090398) **97 genes, 200 annotations.**	GO	Long-term developmental failure
**7. Integrated Stress Response (ISR / ER / UPR)**				
What this reveals: Represents the central stress-integration hub linking protein misfolding, translational repression, stress granule dynamics, and fate enforcement. Prolonged ISR marks transition to DASR and implantation failure.				
**Axis**	**Pathway State**	**GO / Gene Set**	**Source**	**What This Reveals**
ISR / ER Stress	ER stress	response to endoplasmic reticulum stress (GO:0034976) **291 genes, 1029 annotations.**	GO	Protein misfolding burden
ISR / ER Stress	PERK arm	**PERK-mediated unfolded protein response, 22 genes**	MSigDB **MM7288**	Translational shutdown
ISR / ER Stress	IRE1 arm	**IRE1-mediated UPR mouse, 19 genes**	MSigDB **MM7287**	RNA processing stress
ISR / ER Stress	ATF4 / CHOP	**(GO:1990617)** regulation of ATF4 / CHOP **2 genes, 5 annotations.**	GO	Fate enforcement
ISR / ER Stress	Outcome	apoptotic process involved in development (**GO:1902742) 46 genes, 69 annotations.**	GO	DASR / failure
**8. ER Proteostasis / ERAD / Secretory Load**				
**Axis**	**Pathway State**	**GO / Gene Set**	**Source**	**What This Reveals**
ER Stress	Disposal	ERAD pathway (GO:0036503) **1136 genes, 362 annotations.**	GO	Misfolded protein clearance
ER Stress	Regulation	regulation of ERAD pathway (GO:1904292)= GOBP ENDOPLASMIC RETICULUM TO GOLGI VESICLE_MEDIATED TRANSPORT **129 genes**	GO **MM4890**	Acute versus chronic ER stress
ER Stress	Folding capacity	protein folding in ER (GO:0034975)= GOBP PROTEIN FOLDING IN ENDOPLASMIC RETICULUM **6 genes**	GO **MM7021**	ER folding throughput
ER Stress	Trafficking	positive regulation of proteasomal protein catabolic process (GO:1901800) **121 genes, 235 annotations.**		Secretory bottleneck
ER Stress	Outcome	ER-nucleus signaling pathway (GO:0006984) **52 genes, 137 annotations.**	GO	Sustained transcriptional stress
**9. Mitochondrial Stress**				
**Axis**	**Pathway State**	**GO / Gene Set**	**Source**	**What This Reveals**
Mito Stress	ATP production	oxidative phosphorylation (GO:0006119) **146 genes, 310 annotations.**	GO	Respiratory capacity
Mito Stress	Translation	mitochondrial translation (GO:0032543)= GOBP MITOCHONDRIAL TRANSLATION, **127 genes,**	GO **MM6613**	Repair of ETC complexes
Mito Stress	Stress signaling	mitochondrial unfolded protein response (GO:0034514)= **2 genes 4 annotations.**	GO	Retrograde signaling
Mito Stress	Dynamics	mitochondrial fission (GO:0000266) **54 genes 130 annotations.**	GO	Structural adaptation
Mito Stress	Outcome	mitophagy (GO:0000422) **109 genes, 228 annotations**	GO	Quality control
**10. ROS Escalation**				
**Axis**	**Pathway State**	**GO / Gene Set**	**Source**	**What This Reveals**
ROS Escalation	ROS burden	reactive oxygen species metabolic process (GO:0072593) **259 genes, 581 annotations.**	GO	Oxidative load
ROS Escalation	Regulation	regulation of reactive oxygen species metabolic process (GO:2000377) **163 genes, 291 annotations.**	GO	Detoxification capacity
ROS Escalation	Damage	DNA damage response (GO:0006974)	GO	Genotoxic transition
ROS Escalation	Outcome	apoptotic mitochondrial changes (GO:0008637) **126 genes, 314 annotations.**	GO	Failure commitment
	Recovery	mitophagy (GO:0000422) **109 genes, 228 annotations**	GO	Post-injury adaptation
**11. Aerobic Glycolysis ↔ OXPHOS Switching**				
**Axis**	**Pathway State**	**GO / Gene Set**	**Source**	**What This Reveals**

Metabolic State	Pre-stress	glycolytic process (**GO:0006096**) **121 genes, 281 annotations.**	GO	Aerobic glycolysis supporting rapid proliferation
Metabolic State	Regulation	regulation of glycolysis (**GO:0006110**) **65 genes, 117 annotations.**	GO	Control of carbon flux
Metabolic State	Stress-induced shift	tricarboxylic acid cycle (**GO:0006099**) **35 genes, 81 annotations.**	GO	Shift toward ATP-efficient oxidation
Metabolic State	Compensation	oxidative phosphorylation (**GO:0006119**) **146 genes, 310 annotations.**	GO	Increased ATP per carbon (Cantley principle)
Metabolic State	Outcome	pyruvate metabolism (**GO:0006090**) **155 genes, 372 annotations.**	GO	Routing carbon to mitochondrial oxidation
