# Supplementary material for: Systematic Methods to Resolve Lineage-Specific Stress States in Early Mammalian Embryos and That May Enable Miscarriage Prediction
Source: Cells. 2026 May 28;15(11):996. doi: 10.3390/cells15110996 (PMC13256741; doi:10.3390/cells15110996)
Supplement: Supplementary file 1 [file cells-15-00996-s001.zip › cells-4273843 0513 Supplemental Table S3 Expanded PrE VE PE marker panels with citations 04-30-2026.pdf]

Supplemental Table S3. Expanded Gene Sets to Distinguish PrE vs VE vs PE (Mouse-focused)

Purpose: provide higher-specificity marker panels for distinguishing primitive endoderm (PrE), visceral endoderm (VE), and parietal endoderm (PE) in scRNA-seq and bulk RNA-seq. Markers were assembled from peri-implantation single-cell atlases and lineage-transition analyses (see numbered references).

PRIMITIVE_ENDODERM_EARLY_MOUSE (PrE-early / hypoblast-like)

Gene #	Gene Symbol	Notes / evidence (citation #)
1	Pdgfra	[1,2,3]
2	Fgfr2	[1,2,3]
3	Gata6	[1,2,3]
4	Gata4	[1,2,3]
5	Sox17	[1,2,3]
6	Sox7	[1,2,3]
7	Dab2	[1,2,3]
8	Lrp2	[1,2,3]
9	Cubn	[1,2,3]
10	Amn	[1,2,3]
11	Aplnr	[1,2,3]
12	Klf5	[1,2,3]
13	Epcam	[1,2,3]
14	Hesx1	[1,2,3]
15	Dram1	[1,2,3]
16	Bag2	[1,2,3]
17	Creb3l2	[1,2,3]
18	Fam134b	[1,2,3]
19	Col4a2	[1,2,3]
20	Aqp8	[1,2,3]
21	Clic5	[1,2,3]
22	Tmem51	[1,2,3]
23	Gm13270	[1,2,3]
24	Pdzd3	[1,2,3]
25	Chn2	[1,2,3]
26	Timd2	[1,2,3]
27	Clic6	[1,2,3]
28	Havcr1	[1,2,3]
29	P4ha2	[1,2,3]
30	Npepl1	[1,2,3]
31	Leprel1	[1,2,3]
32	Fam176a	[1,2,3]
33	Tmem98	[1,2,3]
34	Dnajc22	[1,2,3]
35	Akr1c19	[1,2,3]
36	Clic3	[1,2,3]
37	Lgals2	[1,2,3]
38	Slc35f5	[1,2,3]
39	Galnt3	[1,2,3]
40	Cdh6	[1,2,3]

VISCERAL_ENDODERM_MOUSE (VE / yolk sac-like maturation)

Gene #	Gene Symbol	Notes / evidence (citation #)
1	Afp	[1,4,8]
2	Ttr	[1,4,8]
3	Apoa1	[1,2,4,5,8]
4	Apoa2	[1,2,4,5]
5	Apob	[1,2,4,5]
6	Cubn	[1,2,4,5,8]
7	Amn	[1,2,4,5,8]
8	Lrp2	[1,2,4,5]
9	Hnf4a	[1,4]
10	Hhex	[1,2,4,5]
11	Foxa2	[1,2,4,5]
12	Krt8	[1,2,4,5]
13	Krt18	[1,2,4,5,8]
14	Krt19	[1,2,4,5]
15	Cer1	[1,2,4,5]
16	Lefty1	[1,2,4,5]
17	Dkk1	[1,2,4,5]
18	Spink1	[1,2,4,5]
19	Slc2a2	[1,2,4,5]
20	Vil1	[1,2,4,5,8]
21	Apln	[1,2,4,5]
22	Cited1	[1,2,4,5,8]
23	Ttr1	[1,2,4,5,8]
24	Tbx3	[1,2,4,5]
25	Foxo4	[1,2,4,5]
26	Hnf1b	[1,2,4,5]
27	Msx1	[1,2,4,5]
28	Tdn	[1,2,4,5]
29	S100g	[1,2,4,5]
30	Igf2	[1,2,4,5]
31	Dusp9	[1,2,4,5]
32	Otx2	[1,2,4,5]
33	Hhex	[1,2,4,5]
34	Plau	[1,2,4,5]
35	Fgfr3	[1,2,4,5]
36	Gata6	[1,2,4,5]
37	Gata4	[1,2,4,5]
38	Gata2	[1,2,4,5]
39	Sox7	[1,2,4,5]
40	Sox17	[1,2,4,5,8]
41	Apoc2	[1,2,4,5]
41	Apoa4	[1,2,4,5,8]
42	Ctsb	[1,2,4,5,8]

PARIETAL_ENDODERM_MOUSE (PE / basement membrane–ECM program)

Gene #	Gene Symbol	Notes / evidence (citation #)
1	Sparc	[7,8]
2	Col4a1	[1,6,7,8]
3	Col4a2	[1,6,7,8]

**4**	**Lama1**	**[1,6,7,8]**
**5**	**Lamb1**	**[1,6,7,8]**
**6**	**Lamc1**	**[1,6,7]**
**7**	**Nid1**	**[1,6,7]**
**8**	**Nid2**	**[1,6,7]**
**9**	**Hspg2**	**[1,6,7]**
**10**	**Fn1**	**[1,6,7]**
**11**	**Col18a1**	**[1,6,7]**
**12**	**Bgn**	**[1,6,7]**
**13**	**Dcn**	**[1,6,7]**
**14**	**Lamb2**	**[1,6,7]**
**15**	**Fgf5**	**[6]**
**16**	**Fgf8**	**[6]**
**17**	**Fgfr4**	**[6]**
**18**	**Sox7**	**[6]**
**19**	**Gata4**	**[6]**
**20**	**Gata6**	**[6,8]**
**21**	**Sox17**	**[6,8]**
**22**	**Klf5**	**[6,8]**
**23**	**Id2**	**[6]**
**24**	**Tead1**	**[6]**
**25**	**Sall4**	**[6]**
**26**	**Tbx3**	**[6]**
**27**	**Elf3**	**[6]**
**28**	**Lin28a**	**[6]**
**29**	**Jun**	**[6]**
**30**	**Klf9**	**[6]**
**31**	**Mycn**	**[6]**
**32**	**Atf6**	**[6]**
**33**	**Atf4**	**[6]**
**34**	**Hopx**	**[6]**
**35**	**Zbtb10**	**[6]**
**36**	**Terf1**	**[6]**
**37**	**Ckap4**	**[6]**
**38**	**Efna3**	**[6]**
**39**	**Klf4**	**[8]**

Numbered References[1-8]

1 Mohammed, H. *et al.* Single-Cell Landscape of Transcriptional Heterogeneity and Cell Fate Decisions during Mouse Early Gastrulation. *Cell Rep* **20**, 1215-1228, doi:10.1016/j.celrep.2017.07.009 (2017).

2 Frankenberg, S. *et al.* Primitive endoderm differentiates via a three-step mechanism involving Nanog and RTK signaling. *Dev Cell* **21**, 1005-1013, doi:10.1016/j.devcel.2011.10.019 (2011).

3 Kang, M., Garg, V. & Hadjantonakis, A. K. Lineage Establishment and Progression within the Inner Cell Mass of the Mouse Blastocyst Requires FGFR1 and FGFR2. *Dev Cell* **41**, 496-510 e495, doi:10.1016/j.devcel.2017.05.003 (2017).

4 Nowotschin, S. *et al.* The emergent landscape of the mouse gut endoderm at single-cell resolution. *Nature* **569**, 361-367, doi:10.1038/s41586-019-1127-1 (2019).

5 Artus, J. *et al.* BMP4 signaling directs primitive endoderm-derived XEN cells to an extraembryonic visceral endoderm identity. *Dev Biol* **361**, 245-262, doi:10.1016/j.ydbio.2011.10.015 (2012).

6 Pham, P. D. *et al.* Transcriptional network governing extraembryonic endoderm cell fate choice. *Dev Biol* **502**, 20-37, doi:10.1016/j.ydbio.2023.07.002 (2023).

7 Mason, I. J., Taylor, A., Williams, J. G., Sage, H. & Hogan, B. L. Evidence from molecular cloning that SPARC, a major product of mouse embryo parietal endoderm, is related to an endothelial cell 'culture shock' glycoprotein of Mr 43,000. *Embo J* **5**, 1465-1472 (1986).

8 Kim, J. *et al.* Neighbor-specific gene expression revealed from physically interacting cells during mouse embryonic development. *Proceedings of the National Academy of Sciences* **120**, e2205371120, doi:doi:10.1073/pnas.2205371120 (2023).
