# Supplementary material for: Systematic Methods to Resolve Lineage-Specific Stress States in Early Mammalian Embryos and That May Enable Miscarriage Prediction
Source: Cells. 2026 May 28;15(11):996. doi: 10.3390/cells15110996 (PMC13256741; doi:10.3390/cells15110996)
Supplement: Supplementary file 1 [file cells-15-00996-s001.zip › cells-4273843 0513 Supplemental Table S4 TSC lineages 4 30 26.pdf]

Supplemental Table S4. Expanded Gene Sets to Distinguish TSC lineages (Mouse-focused)

TSC / Placental lineage GO table, GO-derived functional gene sets relevant to trophoblast stem cell (TSC) lineage progression.

**Functional category**	**MSigDB / GO gene set name**	**GO ID**	**Mouse genes (n) – USER-VERIFIED**	**Definition & lineage interpretation (AmiGO-verified)**
Placental development (broad)	GO_PLACENTA_ DEVELOPMENT	GO:0001890	**201 genes 332 annotations**	*Definition:* The biological process whose specific outcome is the progression of the placenta over time, from its formation to the mature structure. **Interpretation:** Broad placental program; includes trophoblast, vascular, and stromal components; suitable as a high-level parent/background set.
Embryonic placental development	GO_EMBRYONIC_ PLACENTA_ DEVELOPMENT	GO:0001892	**121 genes 176 annotations**	*Definition:* Embryonically driven placental development. **Interpretation:** Mouse-specific placental growth backbone; useful parent comparator for TSC screens.
Maternal placenta Development	GO_ MATERNAL PLACENTA DEVELOPMENT	GO:0001893	**44 genes 59 annotations**	*Definition:* Proliferation of trophoblast cells. **Interpretation:** Functional/state program; not lineage-defining.
Trophoblast giant cell differentiation	GO_ TROPHOBLAST_ GIANT_CELL_ DIFFERENTIATION	GO:0060707	**19 genes 22 annotations**	*Definition:* Differentiation of trophoblast giant cells. **Interpretation:** Terminal TGC fate; useful endpoint readout.
Spongiotrophoblast differentiation	GO_ SPONGIOTROPHOBLAST_ DIFFERENTIATION	GO:0060708	**14 genes**	*Definition:* Differentiation of spongiotrophoblast from unspecialized cells of the ectoplacental cone (EPC). **Interpretation:** Commitment-stage junctional-zone program arising **downstream of TSC via EPC**; may be highly informative for loss of multipotency rather than direct terminal differentiation, depending on gene count.
Glycogen trophoblast differentiation	GO_GLYCOGEN_ CELL_ DIFFERENTIATION	GO:0060709	**1 gene 1 annotation**	*Definition:* Differentiation of glycogen trophoblast cells. **Interpretation:** Correct biology but gene set too small for screening utility.
Trophoblast migration	GO_TROPHOBLAST_ CELL_MIGRATION	GO:0061450	**24 genes 42 annotations**	*Definition:* Migration of trophoblast cells. **Interpretation:** Invasive/behavioral program (EVT-like or invasive TGC), not lineage identity.
